# Precision medicine reaching out to the patients in allergology – a German-Japanese workshop report 

**DOI:** 10.5414/ALX02234E

**Published:** 2021-05-27

**Authors:** Oliver Pfaar, Katharina Blumchen, Eistine Boateng, Eckard Hamelmann, Tomohisa Iinuma, Thilo Jakob, Susanne Krauss-Etschmann, Hiroyuki Nagase, Saeko Nakajima, Taiji Nakano, Harald Renz, Sakura Sato, Christian Taube, Martin Wagenmann, Thomas Werfel, Margitta Worm, Kenji Izuhara

**Affiliations:** 1 Department of Otorhinolaryngology, Head and Neck Surgery, Section of Rhinology and Allergy, University Hospital Marburg, Philipps-Universität Marburg,; 2 Department of Children and Adolescent Medicine, Division of Pneumology, Allergology and Cystic fibrosis, University Hospital Frankfurt, Goethe University, Frankfurt am Main,; 3 Early Life Origins of Chronic Lung Disease, Research Center Borstel, Leibniz Lung Center, Member of the German Center for Lung Research (DZL), Borstel,; 4 Children’s Center Bethel, EvK Bielefeld, University Bielefeld, Bielefeld, Germany,; 5 Department of Otorhinolaryngology, Head and Neck Surgery, Chiba University Graduate School of Medicine, Chiba, Japan,; 6 Department of Dermatology and Allergology, University Medical Center Giessen (UKGM), Justus Liebig University Giessen,; 7 Institute for Experimental Medicine, Christian-Albrechts-Universität zu Kiel, Kiel, Germany,; 8 Division of Respiratory Medicine and Allergology, Department of Medicine, Teikyo University School of Medicine, Tokyo,; 9 Department of Dermatology, Kyoto University Graduate School of Medicine, Kyoto,; 10 Department of Pediatrics, Graduate School of Medicine, Chiba University, Chiba, Japan,; 11 Institute of Laboratory Medicine, Philipps University Marburg, Marburg and Universities of Giessen and Marburg Lung Center (UGMLC), Philipps University Marburg, German Center for Lung Research (DZL) Marburg, Marburg, Germany,; 12 Department of Allergy, Clinical Research Center for Allergy and Rheumatology, National Hospital Organization Sagamihara National Hospital, Japan,; 13 Department of Pulmonary Medicine, University Medicine Essen – Ruhrlandklinik, Essen,; 14 Department of Otorhinolaryngology (HNO-Klinik), Rhinology, Allergy, and endoscopic skull base surgery, Düsseldorf University Hospital (UKD), Düsseldorf,; 15 Department Dermatology and Allergy, Hannover Medical School, Hannover,; 16 Division of Allergology and Immunology, Department Dermatology, Venerology and Allergology, Charité Universitätsmedizin Berlin, Berlin, Germany, and; 17 Division of Medical Biochemistry, Department of Biomolecular Sciences, Saga Medical School, Japan

**Keywords:** allergen immunotherapy, allergic rhinitis, asthma, atopic dermatitis, biologics, insect venom allergy, microbiome

## Abstract

An expert workshop in collaboration of the German Society of Allergy and Clinical Immunology (DGAKI) and the Japanese Society of Allergy (JSA) provided a platform for key opinion leaders of both countries aimed to join expertise and to highlight current developments and achievements in allergy research. Key domains of the meeting included the following seven main sections and related subchapters: 1) basic immunology, 2) bronchial asthma, 3) prevention of allergic diseases, 4) food allergy and anaphylaxis, 5) atopic dermatitis, 6) venom allergy, and 7) upper airway diseases. This report provides a summary of panel discussions of all seven domains and highlights unmet needs and project possibilities of enhanced collaborations of scientific projects.

[Table Abbreviations]

## Introduction

Allergic diseases have shown an increasing prevalence worldwide, including Japan and Germany, throughout the recent decades [1]. Although the reasons for this increase are not yet understood, both genetic and environmental factors are discussed as potential risk factors for the development of allergic diseases. More than 20 years ago, the hygiene hypothesis was developed which implies exaggerated hygiene as a cause for allergic sensitization, while contact with a wide variety of microbes might prevent allergies [2]. Regarding the role of multiple environmental factors as being causal for the development of allergic diseases, there is an important unmet need to better characterize patient-specific risk factors such as nationality, cultural impact, and behavior. Identifying country-specific risk factors and different disease phenotypes would be beneficial to develop appropriate primary and secondary preventive measures for the future [3, 4]. 

Currently, treatment options for IgE-mediated allergic diseases such as allergic rhinitis, atopic dermatitis, allergic asthma, and food allergy typically encompass avoidance measures, (anti-allergic) pharmacotherapy, and allergen immunotherapy (AIT) [5, 6]. 

As a next step, Precision Medicine (PM) has been increasingly investigated for its potential and applicability in treating allergic diseases [3]. The underlying concept has already been established in oncology aiming at individualized care pathways [7]. The fundamental principal behind PM involves multi-sectoral care pathways, patient-centered education, and self-monitoring [3, 4]. Other key elements involve diagnostic pathways for individual phenotyping followed by patient-centered and stratified individualized treatment regimes [4]. An example for the latter are mHealth tools as promising approaches for stratification, indication, and therapy monitoring of AIT in allergic patients [8]. 

The high prevalence of atopic diseases in general as well as the increasing application of PM underline the importance of international exchange of experts in the field aiming to optimize standards in optimal clinical care and to harmonize evidence-based recommendations. Therefore, a group of key opinion leaders from Japan and Germany met and discussed the most unmet needs in the treatment of allergic diseases in order to join forces for the future development of novel precision-medicine approaches. 

The following report summarizes this discussion and comprises seven main sections and related subchapters with key statements from German-Japanese stakeholders in precision medicine in allergology. 

## Materials and methods 

This German-Japanese workshop report has evolved through a multi-step process including in-person and electronic meetings. The initial workshop took place in November 2019 in Frankfurt, Germany, with seven extensive panel discussions of seven key domains. The expert committee discussed current challenges and concomitant guidelines regarding immunology and allergy treatment. After the meeting, panel members from both the Japanese Society of Allergy (JSA) and German Society of Allergy and Clinical Immunology (DGAKI) elaborated extended abstracts on different topics discussed and sent to the corresponding author (OP) who merged them with key points of discussion during the workshop. Hereafter, OP provided an initial draft of this report and circulated this working version to all coauthors for commenting via email. Finally, all coauthors and delegates gave formal approval for submission and publication. 

### Section 1: Basic immunology 

**Parental smoking – consequences for the offspring**

Maternal smoking during pregnancy is an accepted risk factor for impaired fetal growth and lung function, placing the offspring at greater risk of developing asthma and presumably, chronic obstructive pulmonary disease (COPD) later in life [9, 10]. So far, a father’s contribution to offspring health has been deemed to occur purely via inheritable changes in the DNA sequence whereas paternal environmental exposures were considered irrelevant for the health of his children. Recently, it was indicated that paternal health-related behavior can influence the health of children. For example, paternal smoking during his own adolescence was associated with early-onset non-allergic asthma in the offspring [11]. Similar findings were also made for metabolic diseases [12, 13, 14]. 

Seminal fluid and sperm cells are particularly rich in several classes of non-coding RNAs. Among these, microRNAs regulate gene expression post-transcriptionally and thus, modulate cellular functions. Therefore, it has been proposed that sperm cells deliver transfer RNA-derived small RNAs (tsRNAs) into the oocyte which then regulate gene expression post fertilization in the early embryo. Indeed, in a paternal mouse model, a profile of sperm small RNAs have been shown to be different in males on high-fat diet (HFD) as compared to controls [15]. The injection of purified sperm small tsRNAs, 30 – 34 nucleotides, from HFD fathers into normal zygotes recapitulated the paternal metabolic phenotype in male F1 offspring. 

**Establishment of a new mouse model of atopic dermatitis**

Atopic dermatitis (AD) is a chronic inflammatory skin disease with persistent pruritus. Topical corticosteroids and calcineurin inhibitors are used as basic treatments. Moreover, dupilumab, a biologic blocking interleukin (IL-) 4 and IL-13 signals, has recently been commercialized. However, there is still an unmet need to develop a new drug for AD. To do this, a useful mouse model is required. Nunomura *et al.* [16] developed a new mouse model of AD by deleting IKK2, a component of trimeric IκB kinase acting in the canonical pathway of the NF-κB signaling pathway, in facial fibroblasts, but not in the trunk fibroblasts. Due to the spatial distribution of IKK2 deletion, skin inflammation appears only in the face. Moreover, these mice frequently show scratching behaviors responsive to itching. Based on these characteristics, they have been named facial atopic dermatitis with scratching (FADS) mice. FADS mouse showed histological features like AD patients. Type 2 inflammation was dominant, and several members of the IL-10/IL-22 family and the NF-κB family were excessively produced, whereas type 1 and type 17 inflammations were not activated. Indeed, calcineurin inhibitor, a STAT3 inhibitor, and a JAK inhibitor, partially, improved skin inflammation. 

**Allergy-protection with microbes – a feasible approach?**

Asthma is the result of complex gene x-environment interactions. In terms of environmental factors, many clinical and experimental studies indicate the contribution of life-style factors related to urbanization, industrialization, and Western diet [17]. According to the biodiversity hypothesis, such modern living conditions are closely connected to an altered microbiome with reduced diversity and loss of important bacteria involved in tolerance development. In contrast, a biodiverse environment contains microbes which promote health-beneficial effects due to triggering clinical and immunological tolerance development. Candidate microbes including *Acinetobacter lwoffii, Staphylococcus sciuri,* and *Lactococcus lactis*, have been extensively tested [18] indicating their ability to trigger asthma-protective cellular and molecular pathways in models of experimental asthma. Furthermore, *Acinetobacter lwoffii* serves as a Gram-negative model bacterium and exhibits protective effects even in a transgenerational manner [19]. The development of tolerance is an active process of immune education which is accompanied by low-grade inflammation. Recent data indicate that IL-6 may serve as a suitable biomarker to indicate such a beneficial inflammatory response [19]. This is surprising since IL-6 has been previously connected with pathological and asthma-promoting immune situations. Further studies are needed to formally prove the mechanistic and functional role of IL-6 in this regard. 

### Section 2: Bronchial asthma 

**Phenotypes and endotypes of severe asthma in Japan**

Recently, based on a health insurance database [20], it has been found that the prevalence of severe asthma in Japan was 7.8% and that 2.5% of the continuously-treated asthmatics had severe uncontrolled asthma. The rate of death resulting from asthma and the disease burden appears to be lower in Japan than in other countries, probably because the public health insurance covers the entire population and because of good access to hospitals and specialists. 

The mean body mass index is generally low in Japan. However, the incidence of asthma and the occurence of exacerbations seems to be less likely affected by obesity than in western populations, especially in women. 

Japanese cedar pollen is the most common seasonal allergen during spring, and the prevalence of pollinosis among patients with asthma is 34.8% [20]. Pollinosis is related to exacerbations of asthma during spring. Recent biomarker research in Japan revealed that high serum periostin levels were related to accelerated decline in pulmonary function, late-onset asthma, and aspirin intolerance. 

In a Japanese cohort, 76% of patients had some subphenotypes of type 2 asthma, the prevalence of which seems to be higher in Japan than in western populations [20]. 

**From immunosuppression to immunomodulation**

Asthma is a heterogeneous syndrome with different inflammatory phenotypes. In Germany, ~ 7% of the adult population suffers from asthma [21]. The identification of different phenotypes is especially important in patients with severe disease. This patient population is not adequately controlled with high-dose inhaled corticosteroids and additional controller treatment. Indeed, recent data suggest that in this population ~ 30% require the additional use of oral corticosteroids at least once per year [21]. However, given the plethora of potential side effects, recent international and national guidelines have recommended to evaluate patients for potential antibody treatment [22, 23]. Monoclonal antibodies against immunoglobulin E (IgE), IL-5, interleukin 5 receptor (IL-5R) as well as the alpha subunit of the IL-4 receptor (IL-4Rα) can be used in patients with certain phenotypes of severe asthma [24]. These antibodies have the potential to decrease exacerbations, improve lung function and to improve symptoms and quality of life. One of the most important effects of antibody therapy is the decrease in the use of systemic steroids in this patient group. Blocking IL-5, IL-5R as well as IL-4Rα works in patients with increased eosinophil numbers in peripheral blood. Experimental data suggests that IL-5 is important for the increase in all compartments of the body. In contrast, IL-4 and IL-13 in the lung lead to an increased recruitment of eosinophils into the lung [25]. So far it is unclear which of these patients will respond best to either IL-5R blockade or IL-4Rα blockade, and further studies are needed to identify parameters which will help to choose the right antibody for the right patients. 

It is now well recognized, that all anti-inflammatory therapies including antibodies need to be given on a regular basis to remain effective. So far, there is no real disease-modifying treatment available. Different approaches evaluate the use of microbial compounds as potential new disease-modifying therapies [26]. One example is the use of the gastric bacteria *Helicobacter pylori* (*H. pylori*). Epidemiological studies have suggested that people infected with *H. pylori* are protected from the development of asthma [27]. The infection with *H. pylori* reduced the susceptibility to develop allergic airway disease in murine models, and this effect has been linked to the induction of regulatory T cells [28]. The suppressive effect of *H. pylori* infection has been linked to an epigenetic modification of the forkhead box P3 (FOXP3) locus of regulatory T cells. Recent studies have shown that the protective effects of *H. pylori* transmits from infected mothers to their children [29]. In addition, it has been shown that for the protective effect live infection is not necessary. Indeed, lysate of *H. pylori* given systemically has been shown to reduce allergic airway inflammation in a more therapeutic model [30, 31]. These findings further support the investigation of microbial compounds as potential novel treatment options in asthma. 

### Section 3: Prevention of allergic diseases 

**Risk factors of pediatric allergic diseases – insights from birth cohort study in Chiba, Japan**

Environmental factors such as climate, diet, and lifestyle are quite different between Germany and Japan. Risk factors for allergic diseases might be different in these two countries. In the high-risk birth cohort study in Japan (CHIBA study), it has been found that not only eczema before 6 months of age and maternal allergic diseases but also breastfeeding before 4 months of age was a risk factor for egg-white sensitization at 1 year of age [32, 33]. However, the mechanism through which breast milk induces allergic sensitization is currently unknown. Breast milk contains many immunologically active substances, which might be important for sensitization. Also, breast milk contains only minimal amounts of vitamin D, which is thought to have a protective effect against allergic diseases. As a consequence, an interventional birth cohort study using a vitamin D supplement (D-PAC study) has been initiated in Chiba as well as trials on the micro-RNAs in breast milk aimed to better identify the role of miRNA in breast milk in the development of allergic diseases. 

**Primary prevention – what can really be recommend?**

One child of four develops already one form or another of an allergic disease today. A cross-sectional study in German children and adolescents showed that more than 30% of all children between 10 and 17 years have increased levels of specific IgE, and 8% suffer from bronchial (almost always allergic) asthma [34]. The “atopic march” starts as early as at 3 months of age, normally with AD, often followed by signs and symptoms of food allergy (FA). In children with early wheeze, parental atopy and early perennial sensitization are major risk factors for the development of asthma; in infants without early wheeze, parental atopy and early AD are the main risk factors. 50% of children suffering from severe AD develop asthma and 75% develop allergic rhinitis. Primary prevention of allergy, AD, and asthma therefore is a major task [35]. 

After encouraging results from pilot studies on basic skin care for prevention of AD, recent large trials showed conflicting outcomes [36]. It is yet too early to discard basic skin care as a means for primary prevention, and future trials have to address pivotal issues such as optimal product contents, best suitable target population, inclusion criteria aiming to comprise the infants with highest risk, and optimal timing of intervention. 

In a trial in children with single parental atopy history, oral application of bacterial lysates between the 2^nd^ and 7^th^ month of life prevented the development of AD by 20%, but was ineffective in children with dual parental history [37]. This shows very well the limitation of primary prevention in high-risk children with a high genetic burden. 

The Learning Early about Peanut Allergy (LEAP) trial elegantly proved the effectiveness of early introduction of peanuts for the prevention of peanut allergy [38]. This trial however was performed in patients already showing signs of atopic diseases and/or allergic sensitization. The extrapolation of these results to other food allergens failed [39]. The only other positive trial on early introduction successfully prevented the development of hen’s egg allergy in already sensitized children. However, the results demonstrate that early interaction between allergen and immune system is required to introduce immunologic and clinical tolerance. 

Recent trials attempting to convert this into a strategy for preventive AIT showed conflicting results [40]. So far, this approach cannot be recommended as a general tool for primary allergy or asthma prevention. However, in cases of rhinitis patients not yet suffering from asthma, grass AIT for 3 years was effective to reduce the risk of asthma development [41]. 

What can really be recommend for primary prevention [42]?

- Vaginal delivery instead of Cesarean section,
- breast milk for at least 4 months,
- early introduction of solid foods,
- regular vaccination,
- avoidance of:
  - smoking and environmental tobacco smoke,
  - obesity,
  - exposure to traffic emission, pollution, and molds.

What might be successful in the future?

- Prebiotics/probiotics, especially in high-risk children,
- skin barrier enforcement,
- early introduction of food allergens,
- preventive allergen immunotherapy.

### Section 4: Food allergy and anaphylaxis 

**Food allergy – lessons learnt from the anaphylaxis registry**

Anaphylaxis is the most severe manifestation of an allergic reaction with a potential life-threatening outcome. Data about the frequency, clinical reaction patterns, elicitors, reaction circumstances, patient characteristics, and treatment are required to increase awareness but also patient management. The anaphylaxis register is an web-based network, where information as described above is collected by an online questionnaire in a standardized manner. Currently more than 130 centers throughout Europe are participating in the anaphylaxis registry, which is organized as a non-profit organization (network for online registration of anaphylaxis (NORA) e.V.). Within the last 10 years, data from more than 10,000 patients have been obtained. 

The data have not only revealed important information about age-dependent elicitor profiles [43, 44, 45] but also on risk factors of this rare condition. These include age, mastocytosis, exercise, but also the elicitor itself as indicated by using logistic regression analysis. In terms of treatment, the data from the anaphylaxis registry have shown that the current national, but also international, guidelines are not followed to treat anaphylactic patients. Only up to 30% of patients receive intramuscular adrenalin, although this percentage should reach 100% as by definition the patients had respiratory and/or cardiovascular symptoms [46]. These findings demonstrate the need to increase the awareness about the disease and its optimal treatment by implementing educational measures for patients but also doctors. 

Current research in anaphylaxis aims to identify novel biomarkers in anaphylaxis [47]. These shall help to better and faster diagnose the disease, but may also support a better risk assessment regarding repetitive and severe reactions as reflected by different endotypes. 

**Active management of food-induced anaphylaxis (German view)**

The rising trend in the prevalence of food allergy is seen as the third wave of the “allergic epidemic”. Multiple challenges within the diagnostic work-up and management of food-allergic patients and the lack of approved therapies still have to be faced. However, the tackling of these challenges might vary between Germany and Japan. Some aspects of the German guidelines and views were presented to be discussed with the auditorium:

1. Although the gold standard for the diagnosis of food allergy is the oral food challenge (OFC), certain cut-off levels in IgE measurements may be of value to spare this time-consuming and expensive diagnostic tool. Multicenter German trials showed e.g. a 90 – 95% positive predictive value for diagnosing a systemic peanut allergy if Ara h2 IgE is > 42kU/L [48], for a systemic hazel nut allergy if Cor a 14 IgE is > 48kU/L, and for a systemic cashew nut allergy if Ana o 3 IgE is > 2kU/L in children [49].
2. The re-evaluation for diagnosing spontaneous tolerance development to food allergens in children may also vary between Germany [50] and Japan [51]. German decision making is driven by data suggesting that a decrease in wheal size of the skin prick test (SPT) may be associated with tolerance development whereas an increase almost always predicts persistent allergy. It is also driven by epidemiologic data suggesting that milk and egg allergy are outgrown fast after diagnosis with up to 70 – 90% of patients tolerating these allergens by school age whereas the development of spontaneous tolerance e.g. to peanuts occurs only in up to 20 – 50% of patients. Thus, an evaluation of the current allergic status with another OFC will be conducted after 1 – 2 years after the last reaction/diagnosis in milk allergy, after 2 – 3 years in egg allergy, and after 3 – 4 years in peanut/nut allergy. Once the patient reaches school age, intervals for these re-challenges increase, and the timing of a re-challenge will be driven by the occurrence of a decrease in sensitization.
3. German/European guidelines [50] for conducting OFC seem to differ from the Japanese guideline [51] in the aspects of stopping rules, protein doses (e.g., starting dose of 3 mg peanut protein in Germany vs. 190 mg in the Japanese guideline), and time intervals between doses (20 – 60 minutes).
4. The recommendation concerning the strictness of allergen avoidance for the food-allergic child seems to differ between Germany and Japan. For allergists in Germany, the reaction threshold that has been properly diagnosed in a titrated challenge does normally not influence the decision to recommend a strict avoidance of the allergen. This recommendation is driven by the data that thresholds vary from day to day due to augmentation factors [52]. However, the Japanese guideline recommends that strictness of allergen avoidance shall be driven by the reaction threshold under OFC [51]. If a child reacts to a bigger amount of protein during OFC, avoidance of the allergen can be not so strict when compared to a child who reacted e.g. to the first dose of OFC.

Getting to know the different views and experiences on the approaches for the management of food-allergic patients from the other side of the world has broadened the mind of the allergists in both countries. 

**Active management of food-induced anaphylaxis (Japanese view)**

The prevalence of FAs and anaphylaxis has increased in Japan. In 2015, an anaphylaxis registry was established to clarify the clinical course, triggers, and treatment of anaphylaxis in Japan. Approximately 800 patients were included during the study period. The most frequent trigger for anaphylaxis was food, and the top 3 causes were cow’s milk, hen’s egg, and wheat. Oral immunotherapy (OIT) was one of the triggers of anaphylaxis. Although OIT has not been approved as a routine treatment, OIT has been widely performed in Japan. A nationwide survey revealed that more than 100 patients required an adrenaline auto-injector at home during OIT [53]. 

OFCs have become routine practice for the management and diagnosis of FAs in Japan [51]. In the management of food-induced anaphylaxis, half of the high-risk patients passed the OFC targeting a low dose. A stepwise approach for OFC starting with a low dose seems to be useful for avoiding complete elimination of causative foods [54]. Furthermore, low-dose OIT for food-induced anaphylaxis could increase the threshold of anaphylaxis and may lead to higher safety than conventional OIT [55]. 

Although food is an important trigger of anaphylaxis, active management using a stepwise OFC and low-dose OIT may be effective for preventing anaphylaxis. 

### Section 5: Atopic dermatitis 

**Possible role of skin microbiome in the pathogenesis of atopic dermatitis**

The skin is not only a primary site of exposure to pathogens but is also home to a diverse microbiota residing on the surface [56]. The resident skin microbiota can also modulate skin immune cells during homeostasis and play a protective role during infection [57]. 

*Staphylococcus aureus* (*S. aureus*) is frequently isolated from AD patients [58]. Many studies revealed that *S. aureus* promotes inflammation via bacterial penetration across disrupted barrier, that is, skin inflammation in AD accelerates the colonization of *S. aureus* and vice versa. However, the effect of *S. aureu*s colonization on uninflamed skin remains unclear. 

In a skin commensal association model, *S. aureus* is topically applied to murine ears without causing any inflammation. Of note, *S. aureus*-colonized skin showed slight infiltration of neutrophils in the epidermis together with elevating interleukin 1β (IL-1β) mRNA expression. Furthermore, *S. aureus*-colonized skin showed stronger irritant contact dermatitis responses compared to non-colonized skin. Neutrophil depletion suppressed the exacerbated inflammation by *S. aureus* (data on file). 

**New insights into the immunology and possible treatment modalities in atopic dermatitis**

Research on the causes and pathophysiology of AD during the last 30 years revealed that disturbances can be observed in the regulation of skin barrier, the (skin) immune system, and the microbiome on the skin. A hallmark finding was the detection of filaggrin loss-of-function mutations leading to skin barrier defects in ~ 30% of all patients with AD. Meanwhile, a number of proteins involved in the establishment of the skin barrier are known to show mutations in subgroups of patients with AD [59]. The immune system is mainly polarized towards a type 2 cytokine pattern which offers multiple targets for innovative treatments – dupilumab, a blocking antibody of the IL-4 Rα chain is the first substance approved in 2017, and real-world data coming from the German Registry of AD (“TREATgermany”) nicely confirm efficacy data from placebo-controlled studies [60, 61]. A couple of new developments will follow (e.g., anti-IL-13 antibodies, anti-IL-31-receptor antibodies and Janus kinase (JAK) inhibitors acting on the intracellular sites of cytokine receptors) [62]. Proof-of-concept studies point to a possible role of the histamine 4 receptor in AD which may also serve as a target structure for future treatments [62, 63, 64]. A polarized type 2 cytokine pattern is associated with an antibody switch towards IgE which leads to multiple sensitizations to food, environmental, microbial, and even auto-antigens in AD [65]. Allergen-specific IgE has been shown to be clinically relevant for the exacerbation or worsening of dermatitis/eczema in challenge tests in AD patients [66, 67], and there is an ongoing discussion on the value of AIT for the treatment of AD [68]. Finally, the skin microbiome is heavily disturbed in lesional skin of AD with an increase of copy numbers of *S. aureus* and a decrease of bacterial diversity [69]. Of note, anti-inflammatory therapies leading to a better skin condition will also restore the microbial integrity of the skin. 

### Section 6: Venom allergy 

**Molecular diagnostics in hymenoptera venom allergy – benefits and limitations**

The diagnosis of Hymenoptera venom allergy (HVA) is based on the clinical history of allergic sting reactions, skin testing, and laboratory diagnostics for the identification of venom specific IgE (sIgE) directed against whole venom preparations or individual venom allergens [70, 71, 72, 73]. Over the last decade, venomic analysis using classical as well as novel proteomic and transcriptomic approaches has allowed the characterization of individual allergens in several insect venoms [74]. To date, 76 Hymenoptera venom components have been officially listed as allergens (www.allergen.org). In the past, serological diagnostics in Hymenoptera venom allergy was based on the detection of sIgE to whole venom preparations. This conventional sIgE diagnostics is hampered by a high degree of cross-reactivity due to CCD reactivity or peptide-based homologies. The molecular characterization and heterologous production of some of these venom components allowed the rational design of panels of individual allergens for component-resolved diagnosis of Hymenoptera venom allergy. The identification of a number of marker allergens in honey bee (HB) and yellow jacket (YJ) venom (V) (such as Api m 1, Api m 3, Api m 4 Api m 10, and Ves v 1, Ves v 5) has significantly improved the precision of sIgE diagnostics that now can be used to discriminate in most cases genuine HBV and YJV sensitizations [75, 76, 77, 78]. This allows for a better patient selection for venom immunotherapy. In addition, the characterization of sensitizations to different allergens allows the description of individual component-resolved sensitization profiles that may allow risk stratification for treatment outcomes in venom immunotherapy. In particular, HBV-allergic individuals display a wide range of different sensitization profiles [78]. In this context, it was of interest that some of the newly identified major allergens in HBV, namely Api m 3 and Api m 10, were reported to be underrepresented or absent in a number of therapeutic preparations. This observation prompted us to ask whether treatment failure in HBV immunotherapy may be associated with certain sensitization profiles. In a retrospective study of VIT-treated HBV-allergic patients, comparison of sIgE levels to HBV and individual allergens identified predominant sensitization to Api m 10 (> 50% of sIgE to HBV) as the best predictor of treatment failure, with an odds ratio 8.44. No such signal was obtained for dominant sensitization to any of the other allergens [79]. Currently, the role of Api m 10 in HBV allergy and tolerance induction during VIT is not fully understood. However, the high prevalence of Api m 10 sensitization (> 50%), the shortage of Api m 10 in widely used therapeutic HBV preparations, and the significant association of dominant Api m 10 sensitization and treatment failure strongly suggest that Api m 10 is a relevant allergen and that this kind of component-resolved diagnosis may be useful for the risk stratification in HBV immunotherapy. Even though prospective studies are still lacking, the clinical implication would be that HBV-allergic patients with dominant sensitization to Api m 10 are at increased risk for treatment failure in HBV immunotherapy and should preferably be treated with HBV preparations demonstrated to contain an adequate amount of Api m 10 sufficient to induce a robust IgG4 response in treated subjects. 

### Section 7: Upper airway diseases 

**Sublingual AIT (SLIT) in Japan**

Allergic rhinitis induced by Japanese cedar pollen is a common disease in Japan, which is predicted to affect more than one-third of the Japanese population. Therefore, SLIT was developed for mites and pollen, the two major allergens in Japan. 

In addition to the guidelines of the international collaboration *Allergic Rhinitis and its Impact on Asthma* (ARIA), Japan has its own guidelines for allergy treatment. The indications for immunotherapy have been established by the Japanese clinical guidelines for allergic rhinitis. AIT is recommended for patients in whom general therapies are ineffective and for those with at least mild disease. AIT is covered by insurance in Japan; therefore, the annual cost of antihistamine treatment is comparable with that of SLIT. Hence, the cost of AIT is different between Japan and Germany and the cases in which AIT is indicated. However, a common problem is that the penetration rate is not satisfactory. 

Although SLIT is highly effective in Japan and worldwide, it has not been popular so far. In terms of clinical research, digital devices may be useful to provide proper medical care, which might aid in elucidating future directions and the social need for SLIT. At present, several aspects regarding the mechanisms of action of SLIT are unclear, which is addressed in preclinical studies. AIT may become more popular if more basic research is conducted to develop short-term administration methods and to enhance the effects. 

**Placebo effects in allergen immunotherapy – chance or burden**

Placebo effects play a major role in all kinds of medical interventions [80] and different diseases [81, 82, 83, 84]. This phenomenon has been scientifically characterized and was first described by Beecher in the 1950s [85]. Based on this pioneer work, several hypotheses for non-specific effects of medical interventions such as patient perceptions and expectations, spontaneous fluctuations of symptom severity, psychocognitive effects (the so-called “Hawthorne effect” and others), regression to the mean of psychometric evaluations, and several others have been developed [86]. In particular, allergic diseases such as allergic rhinoconjunctivitis or allergic asthma are highly affected by this unspecific effect of interventions and interactions between physician and patients [87, 88]. 

Since AIT is currently regarded as the only available disease-modifying treatment option for patients with respiratory allergic diseases [89, 90, 91], control of these unspecific effects in the clinical development programs of products for AIT is of utmost importance. For this reason, it is demanded by the regulatory authorities, such as the European Medicine Agency (EMA) in their current guideline [92], that phase III (confirmatory) trials “*should be perfomed using a randomised placebo-controlled double-blinded design*” for the demonstration of a (clinically relevant) efficacy of the treatment. Indeed, several clinical trials have demonstrated a relevant level of improvement of symptoms in the placebo arm of randomized patients for both application routes of AIT, SLIT, and subcutaneous (SCIT) treatment (reviewed in [93, 94]). One example is a clinical development program on synthetic peptide immuno-regulatory epitopes (SPIRE) in cat-allergic adult patients. While promising results have been found in the dose-range finding phase II trial (in a chamber model) [95], the (confirmatory) phase III under real-life conditions was not able to demonstrate superiority of the SPIRE approach over placebo due to a tremendous placebo effect in the comparator group [96]. 

However, a high variability of these effects has also been found, and the urgent need for a better understanding of the underlying neuro-psychological mechanisms has been emphasized by many experts in the field [4, 97]. Taken together, current evidence underlines that the placebo effect in AIT is of utmost clinical relevance and as such affects both clinical trial programs and clinical routine. Modern analytical methods (including mHealth and artificial intelligence as well as progress in so-called omics technologies) will help providing a better understanding of neuro-immunological, cognitive, and psychological components of the placebo-response in AIT [98]. 

**Allergic rhinitis and chronic rhinosinusitis – focus on local cytokine networks**

Phenotyping chronic rhinitis is a clinically relevant process and can be achieved when clinical history, testing for allergic sensitization (sIgE in serum or skin prick test), and nasal allergen challenge are combined. Local allergic rhinitis should be understood as a subgroup of allergic rhinitis and is defined by negative sensitization tests and a positive reaction to nasal allergen challenge in the presence of nasal symptoms of allergic rhinitis [99]. 

Nasal allergen challenge can also be used to investigate the pathophysiology of allergic rhinitis. This method has been followed to uncover the time course of cytokine release into nasal secretions. Different studies were able to detect an increased release of IL-4 [100], IL-13, IL-5, IL-10, and IL-31 [101] in the late phase after allergen challenge. Furthermore, the release of IL-17, eotaxin-3, IL-33, and the soluble ST2 [102, 103] could be described as well. ELISPOT assays with nasal tissue samples made it possible to study the production of T helper (Th)2, Th1 cytokine, and IgE production in allergic rhinitis. While natural allergen exposure during pollen season lead to an increased production of both Th2 (IL-4, IL-5) and Th1 cytokines (interferon-γ (IFN-γ)), these patterns were different after experimental nasal allergen challenge, where only Th2 cytokine and IgE production were increased [104]. These results help to understand the complex patterns of cytokine production in allergic rhinitis and suggest that chronic allergen exposure could influence the pattern of cytokine production. 

Chronic rhinosinusitis (CRS) is one of the most frequent chronic diseases and leads to relevant reductions in quality of life. Nasal endoscopy is necessary to differentiate between CRS without nasal polyps (CRSsNP) and CRS with nasal polyps (CRSwNP). Despite continuous treatment with intranasal corticosteroids and endoscopic sinus surgery, recurrence of nasal polyps is relatively frequent. Asthma and NSAID-exacerbated respiratory disease (NERD) have been identified as the most relevant risk factors for recurrence after surgery. 

In several studies, type-2 cytokines (IL-4, IL-13, IL-5) and IgE were found to be elevated in nasal polyp tissue [105, 106]. This type-2 pattern of cytokines is not only dominant in the majority of CRSwNP patients but it has also been found correlated to the coexistence of asthma and nasal polyps [107]. This insight and the clinically challenging problem of polyp recurrence and symptom persistence have been the most important drivers to introduce biologicals into the treatment paradigm for CRSwNP. Positive results in phase 3 studies with the IL4Rα blocking antibody dupilumab [108] and the anti IgE-antibody omalizumab [109] have now led to its approval in many countries [110]. Similar studies with mepolizumab (anti-IL-5), and benralizumab (anti-IL-5R) are either ongoing or not yet published. These positive developments open new and clinically relevant treatment options for patients with severe CRSwNP and will lead to a paradigm shift in clinical care pathways. 

## Conclusion 

The aim of the German-Japanese collaboration reported in this consensus statement is to provide an intense scientific discussion of experts with the aim to optimize standard care of allergic patients in both countries. 

This report emphasizes the key role of phenotyping and optimizing treatment strategies in patients suffering from allergic diseases. Recent investigation of mechanisms in the pathogenesis of atopic diseases paved the way for new treatment options and research models. However, further investigation of immunological mechanisms driven by, e.g., the intestinal microbiome should be prioritized as should be better phenotyping in bronchial asthma. Though the influence of lifestyle, cultural differences, and environmental factors on the prevalence between both countries are still not fully understood, primary prevention measures are generally recommended. Another purpose of this international cooperation was to pinpoint the importance of registers to provide real-life data, e.g., on the prevalence of anaphylaxis, but also to implement harmonized protocols in OFCs and treatment schemes. Recent understanding of skin microbiome and proteomic and transcriptomic mechanisms in atopic diseases such as AD or venom allergy has also been extensively discussed and highlighted in this report. In addition, recent developments in understanding and treating upper airway diseases such as allergic rhinitis or chronic rhinosinusitis have been elaborated. 

Taken together, in the course of the international debate, differences between Germany and Japan regarding diagnosis, treatment preferences, and recommendations became apparent. However, this scientific discussion from experts in both countries has emphasized new aspects and challenges in the treatment and research of allergic diseases (keynotes in Table 1). Close cooperation between the two scientific societies will pave the way for 1) further development of optimized strategies of understanding the mechanisms behind the increase of allergic diseases, 2) further improvement in collecting real-life data on prevalences, 3) the translation from omics research into the clinical field, and also 4) the development of new strategies for optimal care. 

## Acknowledgment 

The German Society of Allergy and Clinical Immunology (DGAKI) and the Japanese Society of Allergy (JSA) thank Mrs. Francesca Gehrt, Marburg, Germany, for editorial support. 

To further facilitate the exchange and establishment of research activities in allergy, the next German Japanese bilateral meeting is scheduled in fall 2021 in Tokio and will be supported by the German (WO 541/21-1) and Japanese Research Foundation in collaboration with the DGAKI and the JSA. 

## Funding 

The expert workshop has been funded by the German Society of Allergy and Clinical Immunology (DGAKI) and the Japanese Society of Allergy (JSA). 

## Conflict of interest 

Dr. Blumchen reports grants and personal fees from Aimmune Therapeutics, grants and personal fees from DBV technologies, outside the submitted work.

Dr. Boateng has nothing to disclose.

Dr. Hamelmann has nothing to disclose.

Dr. Iinuma has nothing to disclose.

Dr. Izuhara has nothing to disclose.

Dr. Jakob reports grants, personal fees and non-financial support from Novartis, grants, personal fees and non-financial support from ALK-Abello, personal fees and non-financial support from Allergy Therapeutics/Bencard, personal fees from Allergopharma, personal fees from Thermo Fisher, outside the submitted work.

Dr. Krauss-Etschmann has nothing to disclose.

Dr. Nagase has nothing to disclose.

Dr. Nakajima has nothing to disclose.

Dr. Nakano has nothing to disclose.

Dr. Pfaar reports grants and personal fees from ALK-Abelló, grants and personal fees from Allergopharma, grants and personal fees from Stallergenes Greer, grants and personal fees from HAL Allergy Holding B.V./HAL Allergie GmbH, grants and personal fees from Bencard Allergie GmbH/Allergy Therapeutics, grants and personal fees from Lofarma, grants from Biomay, grants from Circassia, grants and personal fees from ASIT Biotech Tools S.A., grants and personal fees from Laboratorios LETI/LETI Pharma, personal fees from MEDA Pharma/MYLAN, grants and personal fees from Anergis S.A., personal fees from Mobile Chamber Experts (a GA2LEN Partner), personal fees from Indoor Biotechnologies, grants and personal fees from Glaxo Smith Kline, personal fees from Astellas Pharma Global, personal fees from EUFOREA, personal fees from ROXALL Medizin, personal fees from Novartis, personal fees from Sanofi-Aventis and Sanofi-Genzyme, personal fees from Med Update Europe GmbH, personal fees from streamedup! GmbH, grants from Pohl-Boskamp, grants from Inmunotek S.L., personal fees from John Wiley and Sons, AS, personal fees from Paul-Martini-Stiftung (PMS), outside the submitted work.

Dr. Renz reports grants from German Center for Lung Disease (DZL German Lung Center, no. 82DZL00502), grants from Universities Giessen Marburg Lung Center (UGML), outside the submitted work; and Research support:DFG, BMBF, EU, Land Hessen, DAAD, ALK, Stiftung Pathobiochemie, Ernst-Wendt-Stiftung, Mead Johnson Nutritional, Beckman Coulter; Speakers honorarium: Allergopharma, Novartis, ThermoFisher, Danone, Mead Johnson Nutritional, Bencard; Consulting:Bencard, sterna-biologicals (co-founder).

Dr. Sato has nothing to disclose.

Dr. Taube has nothing to disclose.

Dr. Wagenmann reports personal fees from ALK-Abelló, Allergopharma, AstraZeneca, Bencard Allergie, Genzyme, HAL Allergie, Infectopharm, LETI Pharma, MEDA Pharma, Novartis, Sanofi Aventis, Stallergenes, Teva, all outside the submitted work.

Dr. Werfel reports grants and personal fees from AbbVie, Almirall, Galderma, Janssen/JNJ, Leo Pharma, Lilly, Novartis, Pfizer, Regeneron/Sanofi.

Dr. Worm has nothing to disclose.

**Abbreviations Abbreviations:** Abbreviations.

AIT	Allergen immunotherapy
ARIA	Allergic Rhinitis and its Impact on Asthma
AD	Atopic dermatitis
COPD	Chronic obstructive pulmonary disease
CRS	Chronic Rhinosinusitis
CRSwNP	CRS with Nasal Polyps
CRSsNP	CRS without Nasal Polyps
DGAKI	German Society of Allergy and Clinical Immunology
EMA	European Medicine Agency
FADS	Facial Atopic Dermatitis with Scratching mouse
FA	Food allergy
FOXP3	Forkhead box P3
H. pylori	Helicobacter pylori
HFD	High-fat diet
HB	Honey bee
HVA	Hymenoptera venom allergy
IgE	Immuglobulin E
IFN-γ	Interferon γ
IL	Interleukin
IL-4Rα	Alpha subunit of the IL-4 receptor
IL-5R	Interleukin 5 receptor
JAK	Janus kinase
JSA	Japanese Society of Allergy
LEAP	Learning Early about Peanut Allergy
NERD	NSAID-exacerbated respiratory disease
NORA	Network for online registration of anaphylaxis
NSAID	Non-steroidal anti-inflammatory drugs
OFC	Oral food challenge
OIT	Oral immunotherapy
PM	Precision medicine
*S. aureus*	*Staphylococcus aureus*
SCIT	Subcutaneous immunotherapy
SLIT	Sublingual immunotherapy
SPIRE	Synthetic peptide immuno-regulatory epitopes
SPT	Skin prick test
sIgE	Specific IgE
tsRNAs	Transfer RNA-derived small RNAs
Th	T helper
YJ	Yellow jacket

**Table 1.** Keynotes from different sections.

Section	Key information	Key reference
1. Basic Immunology	The occurrence and severity of respiratory allergies has been shown to be influenced by prenatal and early-life factors. Health-related behavior of both parents and a divers microbiome can promote immunological tolerance.	[19]
2. Bronchial asthma	Despite the efficacy of asthma treatment with monoclonal antibodies in some patients, these therapeutics cannot modulate the immune system and symptoms reoccur after ending therapy. Disease modification might be reached by using microbial compounds treatment.	[26]
3. Prevention of allergic diseases	Lifestyle and environmental factors in Germany and Japan differ and the influence of some remains unclear. However, there is consensus regarding the recommendations for primary prevention for allergy and asthma.	[42]
4. Food allergy and anaphylaxis	Anaphylaxis registers provide important real-life information on causes and risk factors of anaphylaxis. Oral food challenges are seen as gold standard in both countries in food- allergic patients. However, differences in protocols exist and should be further investigated and harmonized. Low-dose OIT might be a future option for food-allergic children at risk for anaphylaxis.	[50, 51]
5. Atopic dermatitis	Restoring the skin barrier can also improve the skins microbiome and thereby prevent exacerbations of eczema and AD.	[69]
6. Venom Allergy	The identification of marker allergens allows a better patient selection for venom immunotherapy due to novel proteomic and transcriptomic approaches.	[79]
7. Upper airway diseases	Compounds of the type 2 inflammation in chronic rhinosinusitis with nasal polyps (CRSwNP) have been characterized and led to new forms of biological treatment options.	[110]
